# Puberté précoce chez une fillette révélant un corticosurrénalome malin

**DOI:** 10.11604/pamj.2017.27.6.12412

**Published:** 2017-05-02

**Authors:** Ayad Anass, Amina Kili

**Affiliations:** 1 Service d’Hématologie et Oncologie Pédiatrique, Hôpital d’Enfants de Rabat, Maroc; 2 Service d’Hématologie et Oncologie Pédiatrique, Hôpital d’Enfants de Rabat, Maroc

**Keywords:** Corticosurrénalome, puberté, chimiothérapie, masse, Corticosurrenaloma, puberty, chemotherapy, mass

## Image en médecine

Il s'agit d'une fillette de 5ans, hospitalisée pour puberté précoce hétérosexuelle. Elle est née par césarienne, le premier cri était immédiat avec un poids de 4 kg, le développement psychomoteur était normal et pas d'histoire de malignité dans la famille. Le début de sa maladie remontait à l'âge de 4 ans par l'apparition d'une pilosité pubienne progressive, d'une acné, de séborrhée et d'une hypertrophie clitoridienne, une voix grave et développement d'une masse musculaire de type masculin. Ainsi que l'apparition de duvet au niveau de la lèvre supérieure et sur le visage. Le tout évoluant dans un contexte d'agitation et d'agressivité. La palpation abdominale, a trouvé une énorme masse difficile à délimiter, allant de l'hypochondre jusqu'au flanc droit et arrivant jusqu'à l'ombilic. L'examen des organes génitaux externes a noté la présence des grandes lèvres. Absence des petites lèvres et hypertrophie clitoridienne (clitoris pénien) et une pilosité pubienne stade 4 selon la classification de Tanner. Le reste de l'examen somatique était normal. Le bilan hormonal a objectivé une élévation de l'aldostérone, Δ Androstendione, 17 hydroxyprogéstérone, SDHA, et la testosterone ainsi qu'une cortisolémie de 8 h à 778,5 nmol/l (VN= 280-876nmol/l). La TDM abdominale a montré une masse d'origine surrénalienne, hétérogène, polylobé avec des zones de nécrose, hyper vasculaires, d'allure maligne corticosurrénalome très probable. Une exérèse tumorale totale a été réalisée avec confirmation anatomopathologique, Les suites opératoires étaient simples. La petite a été mise sous hydrocortisone à la dose 15 mg/m^2^/j associés à un minéralocorticoïde. Aucune chimiothérapie n'a été donnée.

**Figure 1:**
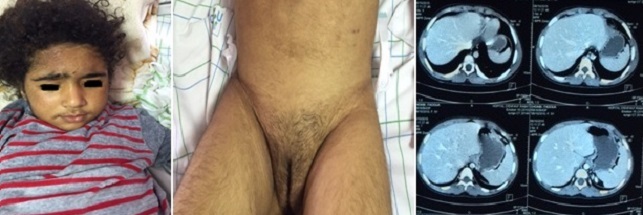
Image de la masse abdominale avec signes de puberté précoce

